# The expanded endoscopic endonasal approach for treatment of tuberculum sellae meningiomas in a series of 40 consecutive cases

**DOI:** 10.1038/s41598-021-83905-7

**Published:** 2021-03-02

**Authors:** Peng Yu, Tutu Xu, Xinyu Wu, Zhitong Liu, Yong Wang, Yibao Wang

**Affiliations:** Department of Neurosurgery, The First Affiliated Hospital of China Medical University, No. 155, North Nanjing Street, Heping District, Shenyang, 110001 Liaoning People’s Republic of China

**Keywords:** Anatomy, Neurology, Oncology

## Abstract

Compared with traditional craniotomy, the expanded endoscopic endonasal approach (EEEA) may have some advantages for tuberculum sellae meningioma (TSM) treatment. We described our experience of the therapeutic effect of endoscopic TSM treatment. From August 2015 to December 2019, 40 patients with a TSM were treated by the EEEA in our institution. EEEA outcome in TSM treatment was analyzed. Among 39 patients with visual impairment, 38 (97.4%) improved their visual function to some extent after the EEEA, and one case had no significant change in visual acuity. Among all patients, 38 (95.0%) achieved gross total resection (GTR) and 2 (5.0%) achieved near-total resection (NTR). Cerebrospinal fluid (CSF) leakage occurred in three patients (7.5%) and meningitis (post-CSF leakage) in two patients (5.0%). Eight patients (20.0%) suffered postoperative hyposmia, three of whom developed long-term hyposmia. One patient (2.5%) suffered from bleeding of the branch of the anterior cerebral artery intraoperatively leading to postoperative acute cerebral infarction. The EEEA is a safe and reliable minimally invasive method for TSM removal. Compared with traditional craniotomy, the EEEA may have better visual outcomes and a higher prevalence of GTR, but carries the risk of CSF leakage.

## Introduction

Meningiomas originating from tuberculum sellae, sphenoid platform and optic chiasmatic groove are collectively called “tuberculum sellae meningiomas” (TSMs), and account for about 5–10% of intracranial meningiomas^1,2^. Surgery is the first-line treatment, but TSMs are located near to multiple crucial neurovascular structures, carrying certain risks and challenges for complete removal^3^. There were several prevailing surgical approaches for TSMs in the past, among which the most classic were pterional, unilateral or bilateral subfrontal craniotomies^4–8^. In recent years, the indications for an endoscopic transsphenoidal approach have been expanding due to the: introduction of high-definition neuroendoscopy; improvement of endoscopic instruments; progress in the technology and materials of skull-base reconstruction.

Therefore, an endoscopic transsphenoidal approach is no longer limited to treatment of intrasellar lesions, but also includes the expanded endoscopic endonasal approach (EEEA) to manages various lesions in the midline area of the skull base^9,10^. The EEEA means that, not only the sellar floor bone, but also the sellar tubercle and sphenoid platform bone can be removed, then the sellar diaphragm is opened, and suprasellar and parasellar lesions can be dealt with. Here, we describe our experience of TSMs when using the EEEA.

## Materials and methods

### Ethical approval of the study protocol

The Education Committee and Ethics Committee of China Medical University approved the study protocol. Written informed consent was obtained from all participants.

### Patient selection

We reviewed the clinical characteristics and imaging data of patients with a TSM who received EEEA-based treatment at our institution from August 2015 to December 2019. This study cohort consists of forty patients with a meningioma locating tuberculum sellae, sphenoid platform and optic chiasmatic groove, both revealed by preoperative magnetic resonance imaging (MRI), and confirmed by pathology studies postoperatively. All patients were treated with the EEEA, and all by the same surgeon. Among all TSMs included in our study, 39 were World Health Organization (WHO) grade I and 1 was at WHO grade II. The age range of patients was 42–73 (mean, 58.9) years. There were 3 male patients and 37 female patients. All patients were treated by a surgical procedure for the first time.

### Clinical presentation

The first symptoms were visual impairment in 37 cases (92.5%), headache in 2 cases (5.0%), a tumor found upon physical examination in 1 case (2.5%), but 39 cases (97.5%) had visual impairment of different degrees of severity. The visual acuity and field, olfaction and endocrinology were evaluated before and after surgery.

### Imaging

Of all patients, six cases had invasion of a unilateral optic canal, which was confirmed intraoperatively. The maximum diameter of the tumor was 16–38 (mean, 24.1) mm. In 10 patients, the tumor was compressed or wrapped around the internal carotid artery (ICA) on MRI. In 10 patients, the tumor was closely related to the anterior communicating artery complex (ACAC).

### Surgical management

All patients underwent EEEA-based treatment of a TSM. The patients were placed in the supine position with head back-ward 10 to 15°, which was fixed in a Mayfield headrest. All patients did not use neuronavigation. The surgical assistant was responsible for lighting and washing with an endoscope to keep the operative field clear. The surgeon was responsible for inserting the surgical instrument through both nostrils^11^.

**Figure 1:**
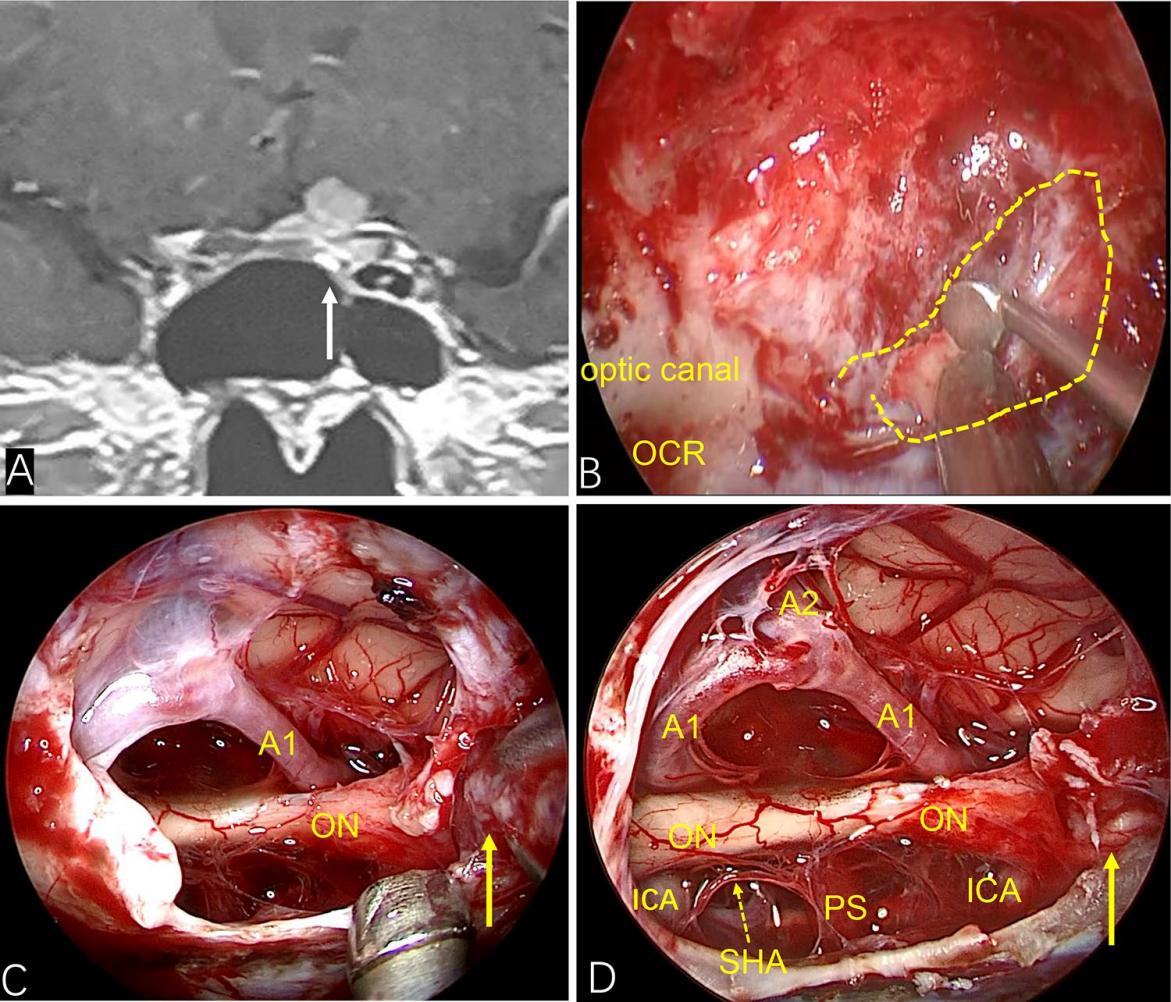
If the tumor invades the optic canal, the optic canal should be fully opened. (**A**) Preoperative coronal MRI showed that the tumor had invaded the left optic canal (white arrow). (**B**) The left optic canal (yellow wire frame) was fully exposed intraoperatively. (**C**) Showing tumors in the optic canal (yellow solid line arrow) (**D**) After the tumor had been removed completely (yellow solid line arrow showed that the tumor in the optic canal had been removed), the optic nerve and its blood-supplying vessels were well protected.

None of our patients had turbinectomy or ethmoid sinus resection. After entering the sphenoid sinus cavity, the sphenoid sinus separation was removed. The ICA position was monitored by vascular ultrasound intraoperatively. The opticocarotid recess (OCR), tubercle of sella, sphenoid platform, anterior sulcus of the optic chiasm and recess of the clivus were identified carefully. According to preoperative imaging evaluation of tumor diameter/location, part of the sellar floor, tuberculum sellae and part of the sphenoid platform were removed. The two sides were opened to the medial part of the prominence of ICA or medial opticocarotid recess. If the tumor had invaded the optic canal, the latter was also opened (Fig. 1). The base of the TSM was confirmed and electrocoagulation carried out. The blood supply of the tumor was cut-off at an early stage. The dura was incised to expose the tumor. Electrocoagulation of the superior intercavernous sinus and hemostasis were carried out. To reduce tumor volume, the tumor was removed piece by piece at the beginning (i.e., central decompression). Endoscopy was employed to observe the adjacent structures around the tumor so as to free the tumor margin safely. After the tumor had been decompressed fully, the tumor was dissociated strictly along the arachnoid plane at the back of the tumor to prevent the arachnoid attached on the surface of the tumor from causing traction damage to the blood vessels passing through the tumor. If there was adhesion between the tumor and optic nerve and main branches of the anterior cerebral artery (ACA), sharp separation could be employed to dissociate the tumor and important neurovascular structures. Finally, a 0° endoscope was employed to ascertain if there was a residual tumor around on the back of the sellar diaphragm. Special attention was paid to check for a residual tumor in the optic canal and, if so, it was removed to finally achieve gross total resection (GTR) of the tumor.

**Figure 2:**
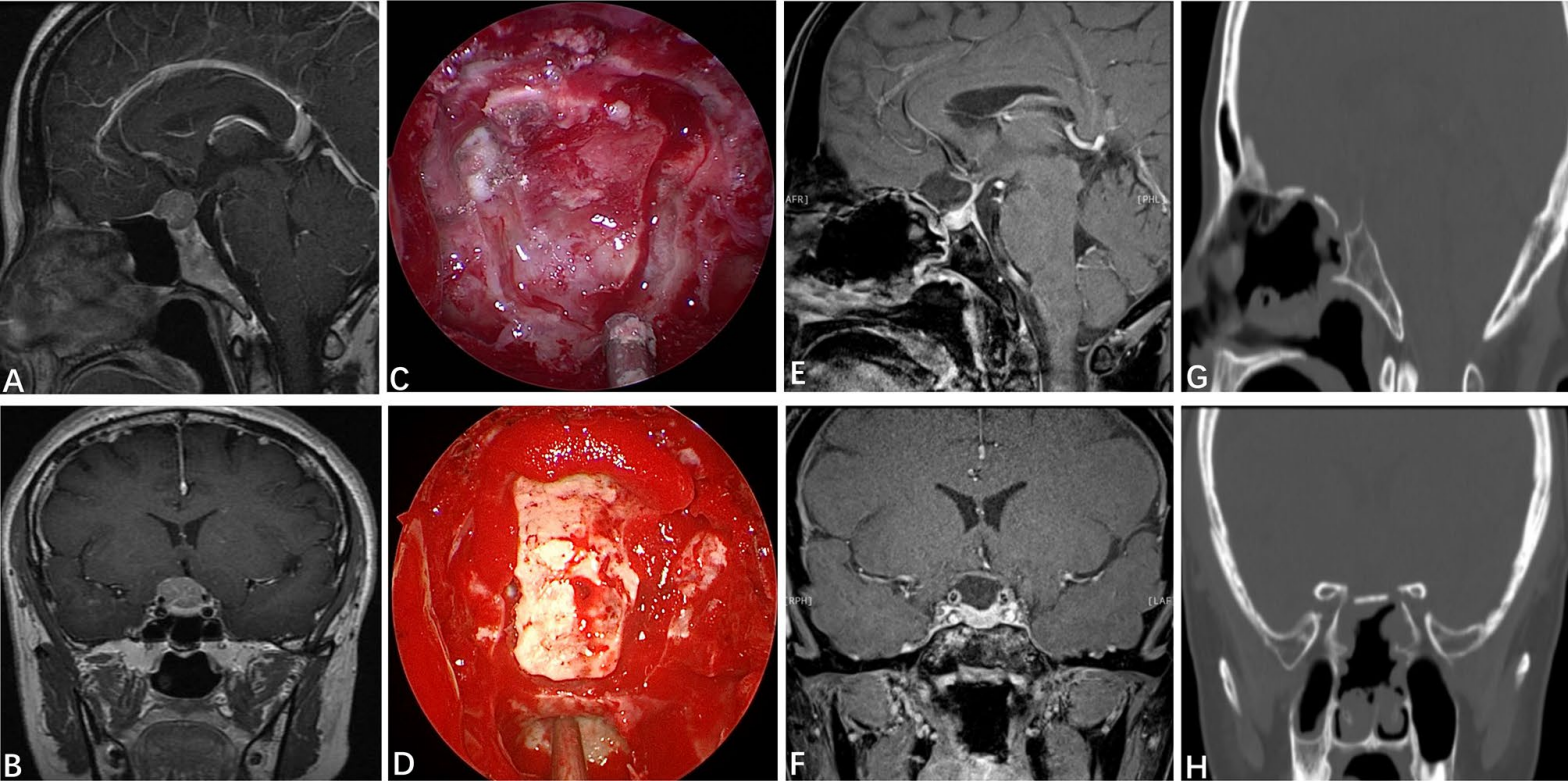
Reconstruction of the skull base with an autogenous in situ bone flap. (**A**,**B**) Preoperative contrast-enhanced MRI of the sellar region indicated that the tumor was to the right, and the structure of the optic nerve was unclear. (**C**) Formation of the autogenous in situ bone flap intraoperatively. (**D**) Autogenous in situ bone flap for skull-base reconstruction. (**E**,**F**) Three months after the EEEA, MRI of the sellar region indicated that the tumor had totally resected, and that the optic nerve, pituitary stalk and pituitary gland were intact. (**G**,**H**) Three months after surgery, CT of the skull base indicated that the bone flap was well restored, and the osseous anatomic structure of the skull base had been restored.

Next, we undertook skull-base reconstruction. The dural defect was inlaid with artificial dura mater. Biological protein glue was sprayed to seal and strengthen the artificial dura mater. The in situ bone flap from the sellar region to the suprasellar region was used for restoration (for some patients whose in situ bone flap was not available, we used autogenous thigh fat and fascia lata to reconstruct the skull base) (Fig. 2). The in situ bone flap or fascia lata was covered with a vascularized nasoseptal flap (VNF), and then biological protein glue was sprayed to seal it. Multilayer reconstruction was undertaken using various materials. Finally, the reconstruction site was fixed with a 14-F Foley balloon catheter^12^.

### Ophthalmological examination and evaluation

Ophthalmological examinations were performed by ophthalmologist of the Ophthalmological Department. All patients were examined for visual acuity, visual field and fundus before the operation and during the postoperative follow-up period, which consisted of visual acuity tested with the correcting glasses for both eyes, Goldmann perimetry used to evaluate visual field defect and funduscopy performed to evaluate pallor or swelling of the optic discs. By comparing preoperative and postoperative visual examination results, the ophthalmologist assessed whether the patient's visual outcome was improved, unchanged, or worse according to the visual impairment score (VIS). The VIS is calculated by summing the reference table scores for visual acuity and visual field defect. Normal vision is graded as 0, and the maximum score of 100 represents blindness^7^.

### Evaluation of surgical results

The clinical outcome of the EEEA for TSM treatment was evaluated by postoperative examination (visual, olfactory, clinical and endocrinologic). At postoperative day (POD)3, MRI of the sellar region was reexamined routinely. According to endoscopic observation intraoperatively and MRI of the sellar region postoperatively, the degree of tumor resection was evaluated. GTR is defined as Simpson grade-1 resection in which no tumor remains: (i) as detected by a 0° endoscope during surgery; (ii) as confirmed by contrast-enhanced MRI of the sellar area after surgery. If GTR could not be achieved, compared with the preoperative tumor volume, the degree of resection was classified: “near-total” if > 90% tumor was removed, “sub-total” if 50–90% was removed, and “partial” if < 50% was removed. Tumor volume was measured by 2 separate radiologists before and after surgery.

### Statistical analyses

We used descriptive statistics to analyze the imaging data, clinical manifestations, complications, and outcomes of patients. Fisher’s test (total sample size n < 40 or theoretical numbers T < 1) was used to compare the GTR prevalence of different tumor characteristics and prevalence of cerebrospinal fluid (CSF) leakage after different methods of skull-base reconstruction. P < 0.05 was considered significant. SPSS v22.0 (IMB, Armonk, NY, USA) was used for analyses.

### Informed consent

Informed consent was obtained from all individual participants included in the study.

## Results

### Clinical outcome

The preoperative VIS of 39 patients with visual impairment were 40–88 (mean, 71.2) and 34–82 (mean, 58.3) postoperative. Among 39 patients with visual impairment, 38 patients (97.4%) improved their VIS to some extent after the EEEA. One patient had no significant change in vision. One patient experienced short-term visual deterioration postoperatively. After corticosteroid impulse therapy the visual acuity recovered to the preoperative level and visual acuity improved after 1-month follow-up. Headache disappeared in two patients at long-term follow-up after surgery. One patient in whom a tumor was found upon physical examination had no obvious symptoms preoperatively and, after receiving EEEA-based treatment, there was no change in vision and no complications were observed. The overall mean length of hospital stay was 6.8 days (range 4–16 days). Postoperative complications (e.g., hypophysis hypofunction, epilepsy) were not observed in any patient. No patient died after EEEA-based treatment.

### Surgical results

**Figure 3:**
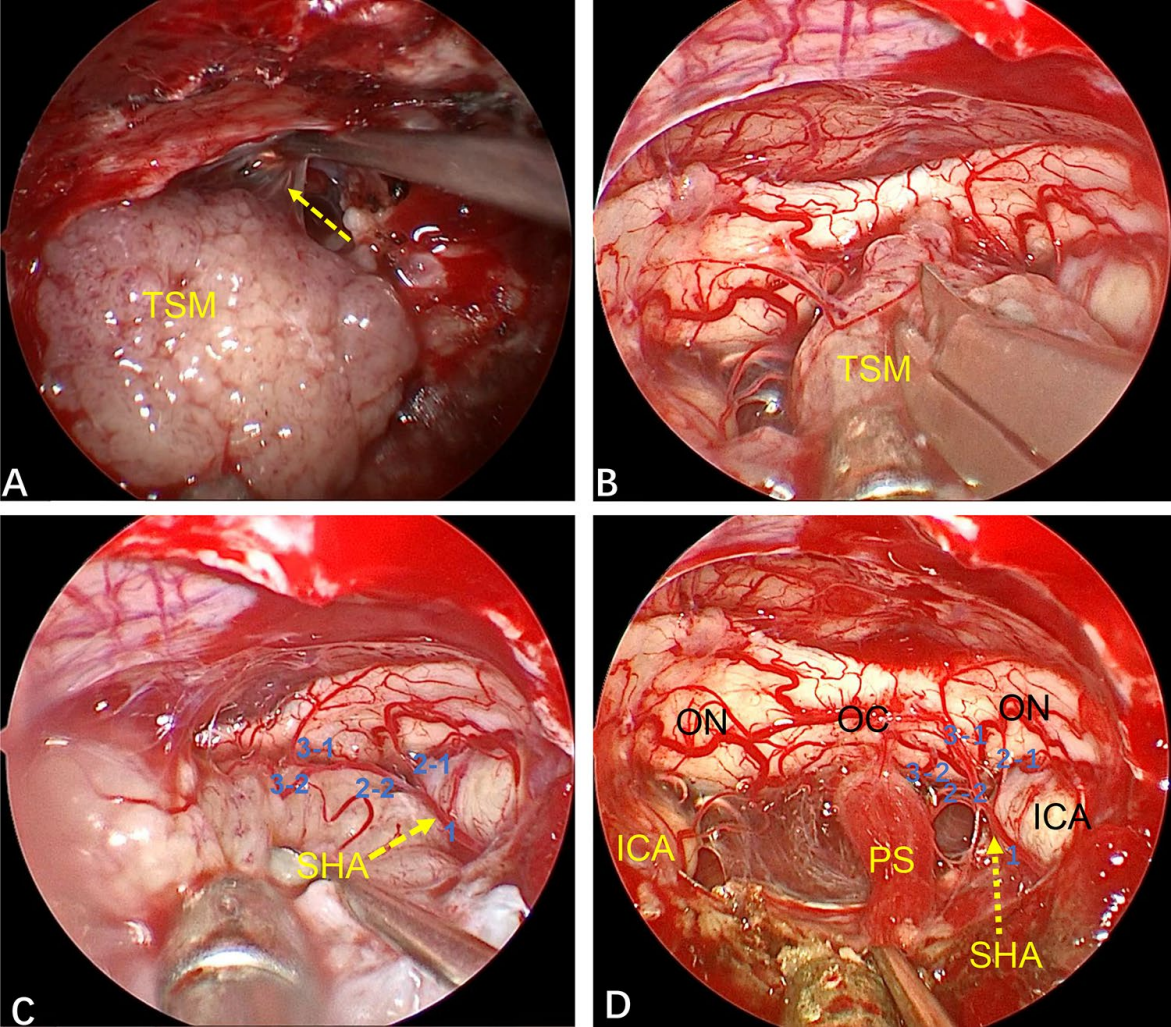
Whether an arachnoid plane is present is closely related to the difficulty of tumor resection. (**A**) This TSM has a complete arachnoid plane (yellow arrow). Strict separation along the arachnoid plane is done during tumor removal, causing less disturbance to neurovascular structures. (**B**–**D**) In another patient, the tumor lacked an arachnoid plane and the branches of the superior hypophyseal artery (SHA) were closely related to the tumor. After the tumor had been removed completely, the SHA branch supplying the optic nerve was well protected (blue Arabic numerals in **C**,**D**).

Among all patients, 38 (95.0%) achieved GTR (Simpson grade I), and 2 (5.0%) achieved NTR. In six patients (15.0%), the tumor invaded the optic canal, and the latter was opened intraoperatively to remove the tumor. Thirty-four patients (85.0%) had a complete arachnoid plane, and six patients (15.0%) lacked an arachnoid plane (Fig. 3).

### Complications and management

**Figure 4:**
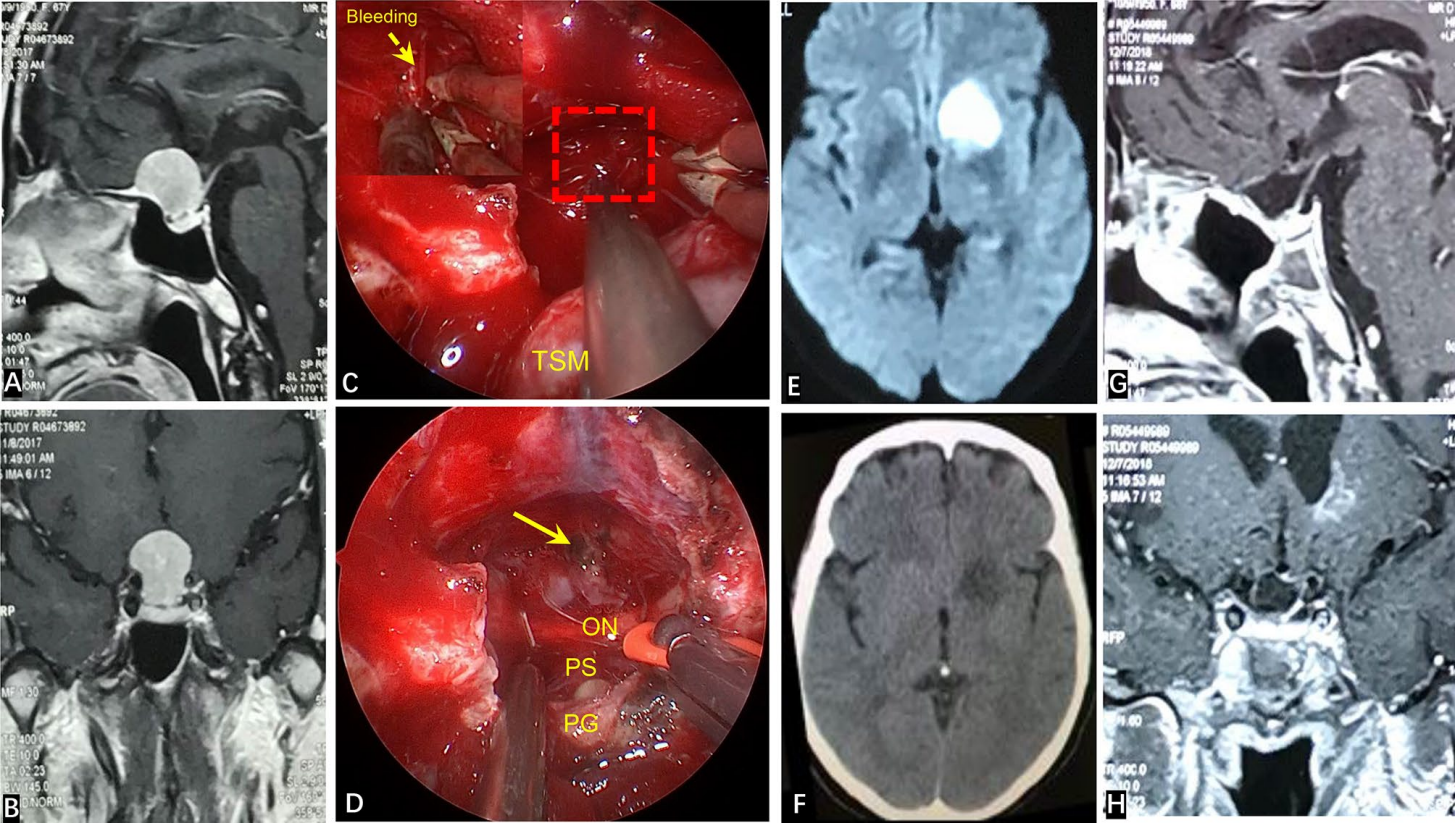
(**A**,**B**) Preoperatively, contrast-enhanced MRI of the sellar area showed the dimeter and shape of the tumor. (**C**) The texture of this tumor was tough, and bleeding occurred in the A2 branch of the ACA intraoperatively (red dotted line box is the bleeding area, and the upper left corner is an enlarged image of the bleeding). (**D**) GTR of the tumor after completion of electrocoagulation and hemostasis of the A2 branch of the ACA (yellow arrow). (**E**) Walking instability occurred at POD5. Diffusion-weighted imaging shows recent infarction of the left caudate nucleus head. (**F**) CT reexamination at POD10 reveals muscle strength returned to normal and the patient could move freely. (**G**,**H**) One year after surgery, contrast-enhanced MRI of the sellar region showed that the tumor had been completely resected, and that there was no sign of residual tumor or recurrence.

CSF leakage occurred in three patients (7.5%) and meningitis (post-CSF leakage) was found in two patients (5.0%). One case was treated with secondary fat repair under endoscopy combined with lumbar drainage, and the two other patients were treated with lumbar drainage. All patients with CSF leakage were treated with antibiotics. After the treatment mentioned above, CSF leakage and infection in these patients were cured. Eight patients (20.0%) had postoperative hyposmia, among which five cases had nasal irrigation in the early postoperative period, four patients had olfactory function return to normal, and one case had significant improvement in olfactory function. Three patients (7.5%) developed long-term hyposmia, and two of them were associated with CSF leakage and infection after surgery. One patient (2.5%) suffered from bleeding of the A2 branch of the ACA intraoperatively. At POD 5, the muscle strength of one side of the lower limb decreased. Diffusion-weighted imaging showed that the caudate nucleus head (CNH) had suffered infarction recently (Fig. 4). After symptomatic treatment, muscle strength returned to normal on POD 30. The complications of all patients are summarized in Table 1.

**Table 1:** Surgical complications.

Complication	Number of cases (%)
CSF leak	3 (7.5%)
Meningitis (post-CSF leak)	2 (5.0%)
Transient visual impairment	1 (2.5%)
Acute cerebral infarction	1 (2.5%)
**Hyposmia**	8 (20.0%)
Short-term	5 (12.5%)
Normal after nasal irrigation	4 (10.0%)
Significant improvement after nasal irrigation	1 (2.5%)
Long-term (no nasal irrigation)	3 (7.5%)
Associated with meningitis	2 (5.0%)
Seizures	0 (0%)
Hypopituitarism	0 (0%)

### Recurrence and follow-up

**Figure 5:**
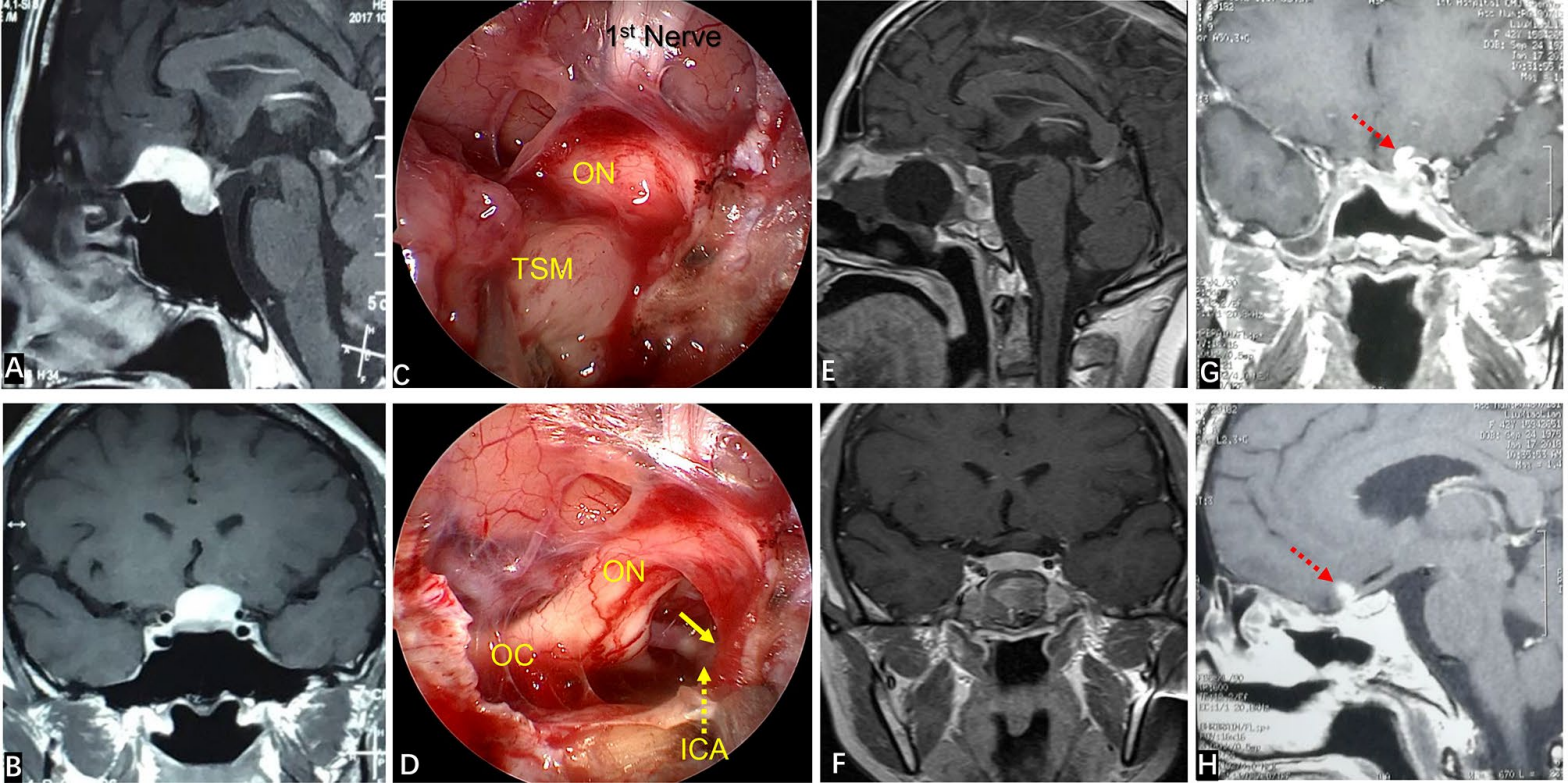
(**A**,**B**) Preoperative contrast-enhanced MRI of the sellar region showed the tumor extending along the sphenoid platform. (**C**,**D**) Intraoperatively, the tumor adhered closely to the left optic nerve and ICA. After tumor removal, little residual tumor remained at the left ICA (yellow solid-line arrow). (**E**,**F**) At POD5, contrast-enhanced MRI of the sellar region showed that the tumor has been resected completely and that no residual tumor remained at the left ICA. (**G**,**H**) Three months after surgery, contrast-enhanced MRI showed that a small tumor mass remained in the left ICA (red dotted-line arrow).

Patients were followed up for 5–57 (median, 15) months. Follow-up comprised MRI of the sellar region, endocrinology evaluation, olfactory examination, as well as clinical and ophthalmic examinations for all patients. Of all patients, only one case (2.5%) had tumor recurrence, at 3 months after surgery. And a small tumor mass remained in the left ICA (although no obvious tumor was found during postoperative MRI review). Postoperative pathology studies and immunohistochemistry indicated that the Ki-67 Proliferation Index was ~ 20%, indicating atypical meningioma (WHO grade II) (Fig. 5). Finally, the patient underwent craniotomy 8 months after the first surgical procedure.

## Discussion

TSMs are closely associated with the optic nerve, which are often compressed. Therefore, the most common symptoms of TSMs are vision/visual-field disorders, so saving vision is the main purpose of TSM treatment^7,13,14^. In addition, the duration of preoperative visual impairment is closely related to the prevalence of postoperative improvement in visual function^7,15,16^. Studies have shown that an untreated meningioma of the skull base tends to continue to grow^17^. Therefore, once the diagnosis is definitive, surgical treatment should be carried out as early as possible.

Regarding the choices of surgical approach for TSM, some authors believe that tumor maximum diameter > 4 cm, lateral extensions, invasion of the optic canal or vascular encasement is more suitable for craniotomy^18,19^. The above are only relative contraindications of EEEA. Considering the endoscopic learning curve, surgeons with rich experience in endoscopy can still try to use EEEA to remove tumors with those features. With the progress of the technology and conception of the professional endoscopic team, EEEA is now more suitable for tumors that invade the optic canal.

Compared with a traditional craniotomy, the advantage of the EEEA is that it can transform deep skull-base tumors into superficial convex tumors, and the objective of the surgical procedure is “coaxial” with the direction of tumor growth, which is more conducive to safe resection of lesions^20^. Simultaneously, the invaded bone and dura mater can be resected, and Simpson grade-I resection can be achieved^21^. A skull-base procedure can deal early with the blood supply of the tumor base, and reduce the risk of intraoperative hemorrhage. Intraoperatively, brain tissue and neurovascular structures are not retracted and the risk of retraction injury is reduced. Meanwhile, the endoscope has a wide perspective, the operative field of view is clear, and local structures are enlarged. Also, multi-angle observation can reduce the possibility of injury to neurovascular structures intraoperatively^10^. The EEEA is also associated with a shorter duration of hospitalization and acceptable cosmetic effects^10,20,22^.

### Visual outcome

Several studies have shown that, compared with craniotomy, the EEEA leads to a higher prevalence of visual improvement upon TSM treatment (Table 2). In 2008, Divitiis et al. reported 51 cases of TSM. Among them, after endoscopic resection of TSMs in seven cases, 71.4% of patients had improved vision, 28.6% of patients had no deterioration of vision. In the transcranial group, postoperatively 61.4% of patients had improved vision, 25% of patients had unchanged vision but, in 13.6% of patients, vision had deteriorated^23^. In 2014, Koutourousiou et al. used the EEEA to treat 75 patients with a TSM. Postoperative visual acuity improved in 85.7% of cases, but deteriorated in 1.3%^20^. In 2018, Doo-sik Kong et al. reviewed 178 patients with TSM, of which 84 underwent EEEA and 94 underwent craniotomy. The improvement rate of visual symptoms in EEEA group is higher than that in craniotomy group (85.0% vs. 55.8%), and the postoperative visual deterioration in EEEA group is less than that in craniotomy group (5.0% vs. 16.9%)^24^. In 2012, Bohman et al. undertook a meta-analysis of the literature. They showed that the prevalence of visual deterioration of patients with TSM after craniotomy and endoscopic transnasal treatment was significant. The prevalence of visual deterioration after craniotomy was ≤ 9.2%, but only 1.3% after endoscopic surgery^25^. Therefore, the visual outcome after using the EEEA is better than that of craniotomy irrespective of improvement or deterioration of vision. We observed a similar phenomenon in our study. Among 39 patients with visual impairment, 38 patients in which the EEEA was employed showed varying degrees of improvement in vision, with improvement recorded in 97.4% of cases.

**Table 2:** Literature review on operative results using various surgical approaches for TSM treatment.

Authors and year [reference number in manuscript]	Number of cases	Surgical approach	GTR, %	Visual outcome, %			Mortality, %
				Improved	Unchanged	Worsened	
Goel et al. 2002^32^	70	Unilateral subfrontal mainly	84.3^a^	62.9	27.1	10	3
Fahlbusch and Schott 2002^7^	47	Pterional	97.9^b^	80	NR	20	0
Divitiis et al. 2008^23^	44	Pterional mainly	86.4^c^	61.4	25	13.6	0
Chokyu et al. 2011^5^	34	Subfrontal	79.4^d^	90.6	9.4	0	0
Doo-sik Kong et al. 2018^24^	94	Pterional mainly	79.8^a^	55.8	27.3	16.9	1.1
Divitiis et al. 2008^23^	7	EEEA	85.7^c^	71.4	28.6	0	14.3
Wang et al. 2010^14^	12	EEEA	91.7^d^	92	8	0	0
Koutourousiou et al. 2014^20^	75	EEEA	81.4	85.7	10.7	3.6	1.3
Doo-sik Kong et al. 2018^24^	84	EEEA	83.3^a^	85	10	5	1.2
Present study	40	EEEA	95.0	97.4	2.6	0	0

*NR* not reported.

^a^No mention of Simpson grading system.

^b^According to intraoperative findings of the surgeon; the Simpson grading system and MRI were not mentioned.

^c^No mention of Simpson grading system and no postoperative MRI review.

^d^Including Simpson grades I and II.

EEEA-based treatment of TSMs has several natural advantages in terms of the visual prognosis. First, TSMs often invade the optic canal (especially the medial wall of the optic canal). Invasion of unilateral or bilateral optic canals has been reported in 67–77% of TSM cases^26,27^. Therefore, even though the TSM is small, the narrow space of the optic canal can result in very severe visual impairment. Also, the TSM can lead to hyperosteogeny of the optic canal, thereby compressing the optic nerve further. The EEEA is relatively simple and convenient for opening the optic canal, and can fully decompress the upper, lower and medial wall of the optic canal^14,20^. The EEEA is more advantageous for exposing the medial wall of the optic canal than the traditional transcranial approach. In the present study, six patients suffered invasion of a unilateral optic canal. Intraoperatively, we fully opened the optic canal and removed the tumor in the optic canal without manipulation of the optic nerve (Fig. 1). Resection of the tumor in the optic canal not only improved visual acuity but also reduced the risk of tumor recurrence.

**Figure 6:**
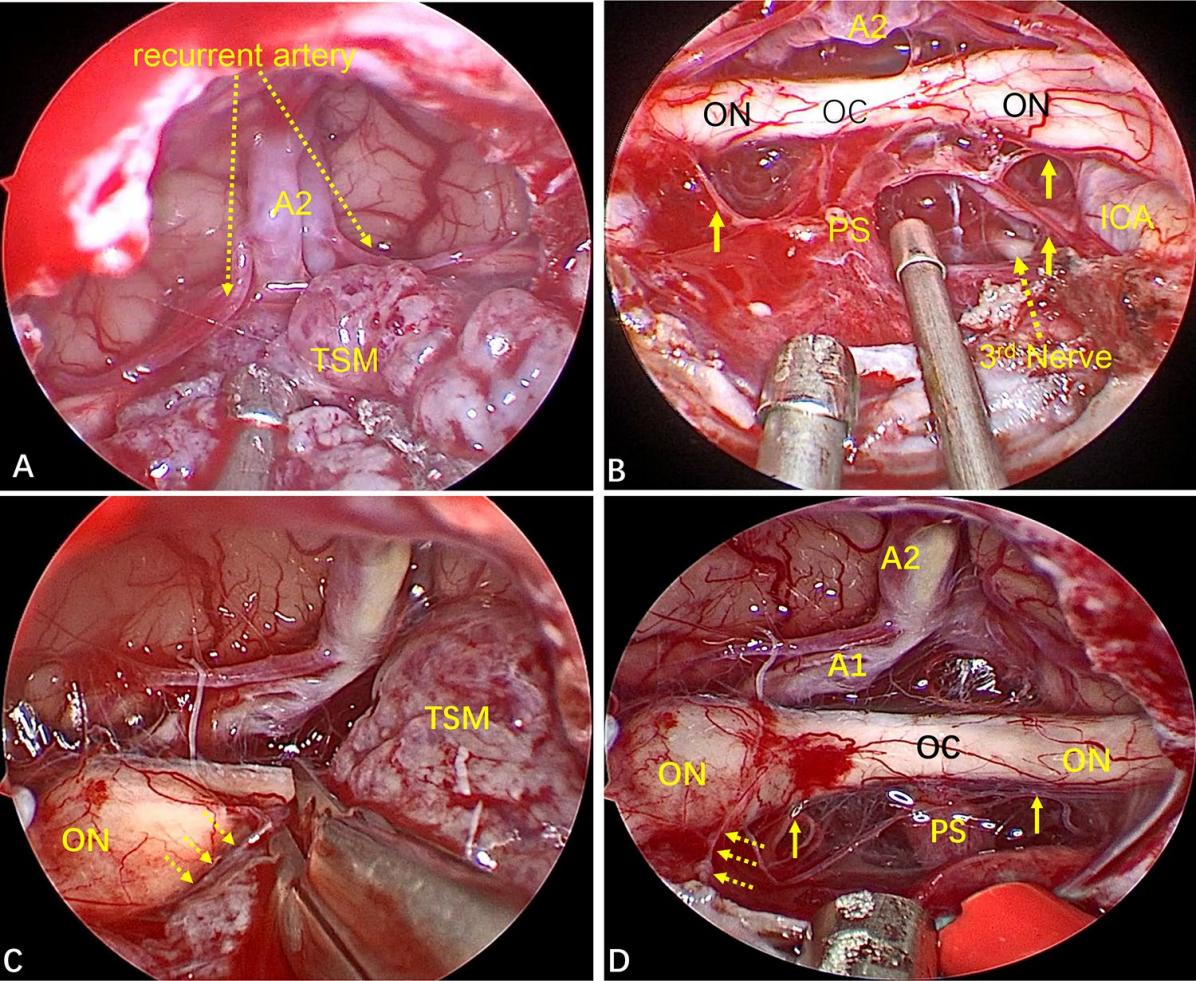
(**A**) Intraoperative image showing the close relationship between the tumor and ACAC. (**B**) The perforating branches of small blood vessels closely related to the optic nerve were fully preserved after total resection of the tumor (yellow solid-line arrow). (**C**) The small vessels supplying the optic nerve were closely related to the tumor (yellow dotted arrow). (**D**) Total resection of the tumor revealed that the right optic nerve suffered tumor invasion. The blood-supplying vessels of the optic nerve closely related to the tumor were well protected (yellow dotted-line arrow), and the perforating branches of other small vessels closely related to the optic nerve were fully preserved (yellow solid-line arrow).

The EEEA provides a bottom–top perspective for TSM treatment, with the surgical perspective being “coaxial” with the direction of tumor growth. The superior hypophyseal artery (SHA) originates from the ICA. Bilateral SHAs send out small branches to supply the lower surface of the optic chiasm, and branches from the ACAC provide the blood supply for the upper surface of the optic nerve^20,28^. Whether these blood vessels are preserved is closely related to the postoperative vision of patients^24^. The EEEA directly displays the neurovascular structure of the suprasellar and infrachiasmatic areas from the bottom. This renders these neurovascular structures fully visible, thereby ensuring that the perforating branches of the small vessels of the optic nerve can be fully preserved (Fig. 6). Second, the bottom–top perspective provides good protection to the neurovascular structures which are behind the arachnoid plane, reducing the need for manipulation of these important structures (Fig. 3A). More importantly, in some patients with a TSM, the arachnoid plane is lacking, and the tumor often adheres to the neurovascular structure to a certain extent. However, the transnasal approach provides a bottom–top visual field. Also, under the direct vision of endoscopy, the operative field is clear, local structures are enlarged, and observation from multiple angles (as long as there is careful separation), even in patients without an arachnoid plane, allows these small vascular perforating branches supplying the optic nerve to be preserved (Fig. 3B–D). Therefore, compared with craniotomy, in EEEA-based treatment of TSMs, the risk of postoperative visual deterioration is minimized^23,25,29,30^. In our study, no visual deterioration occurred in any patient during long-term follow-up.

**Figure 7:**
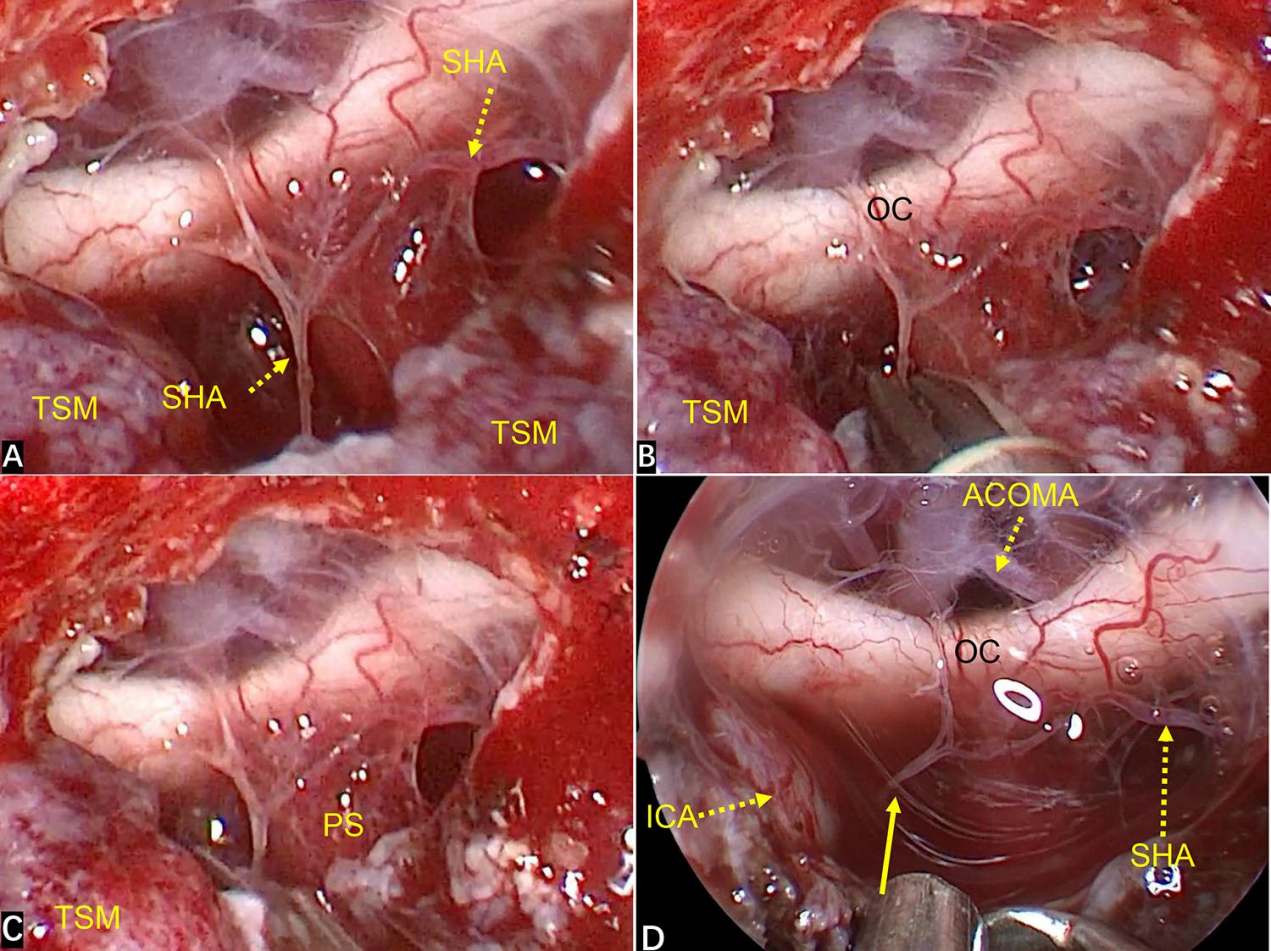
(**A**) Intraoperatively, the right SHA was entangled with the tumor and could not be separated. The left SHA was well developed. (**B**,**C**) The right SHA was cut off to prevent the ICA from tearing and bleeding caused by excessive traction, and the tumor was removed. (**D**) The stump of the right SHA (yellow solid-line arrow) is displayed after GTR.

The SHA originates from the medial wall of the ICA and provides the blood supply on the lower surface of the optic chiasm. If bilateral SHAs are damaged simultaneously, it is likely to cause insufficiency of the blood supply to the optic chiasm and blindness. But whether occlusion of a unilateral SHA can lead to visual impairment is not known. In an anatomic study, Truong et al.^28^ showed that determination of visual outcome after occlusion of the SHA on one side was not possible. However, the overall impression was that the blood supply and vascular anastomosis of the optic nerve and optic chiasm were rich. Hence, it might be safe to disconnect the SHA on one side in a high percentage of cases. This hypothesis is consistent with the results of a large series of studies by Horiuchi et al. on the visual outcomes of clipped SHA aneurysms^31^. Two of our patients had blood vessels from the SHA involved in blood supply to the tumor. In one of these cases, the right SHA was small and entangled with the tumor, which could not be separated. The left side of the SHA was well developed, so we actively cut off the right SHA to prevent the ICA from tearing and bleeding caused by excessive traction, and removed the tumor. The vision of patients after tumor resection was better than that before surgery (Fig. 7). We should try our best to protect the SHA, but if the adhesion between the SHA and the tumor is difficult to be separated and the bilateral SHAs are well developed, it may be safe to cut-off one branch for the convenience of tumor resection.

### Surgical outcome

Several scholars have focused on craniotomy for TSMs (Table 2). Goel et al. described their experience of 70 cases of TSMs. They mainly used a subfrontal approach for resection, and GTR was 84.3%, but the Simpson grading was not mentioned^32^. Fahlbusch and Schott reported on 47 cases of TSM who underwent resected by a pterional approach, and GTR was 97.9%. However, the results were based mainly on the surgeon’s intraoperative findings^7^. Chokyu et al. reported on TSM resection in 34 cases using a subfrontal approach. GTR was 79.4%, but statistical analyses included Simpson grades I and II^5^.

**Figure 8:**
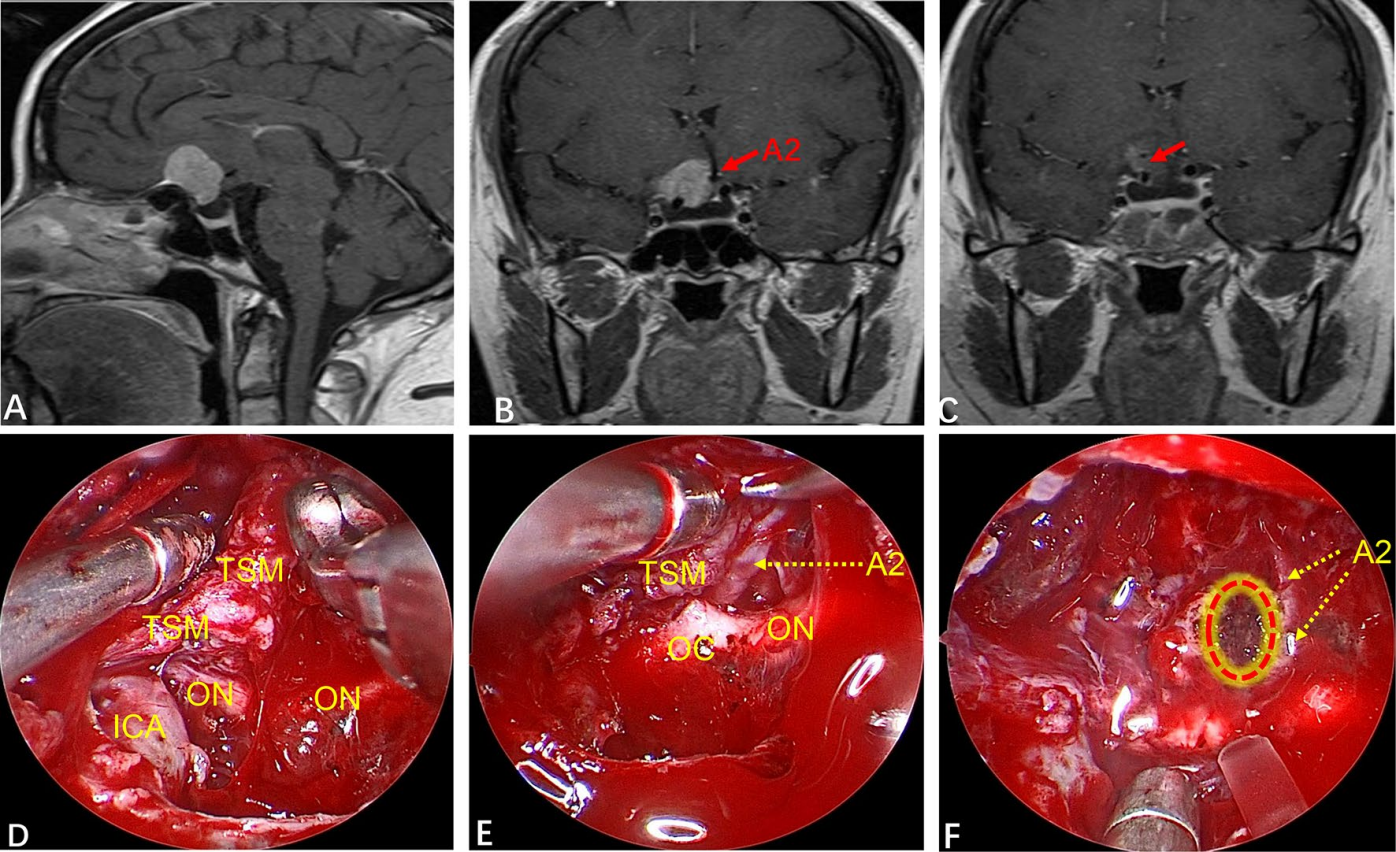
(**A**,**B**) Contrast-enhanced MRI preoperatively indicated that the tumor was closely related to the internal carotid artery and A2 branch of ACA. (**C**) At POD3, MRI reexamination showed the degree of tumor resection. There was no tumor residue at the A2 branch of the ACA. Whether there was a small amount of tumor remaining at the right internal carotid artery (ICA) was not clear (red arrow). (**D**) This intraoperative image shows the tumor mass toughened by fibrosis and beyond the right ICA. (**E**) There was a close relationship between the A2 branch of the ACA and fibrosis-toughened tumors. (**F**) A few tumors were linked inextricably to the A2 branch of the ACA, and residual tumors were electrocoagulated repeatedly intraoperatively (red dotted circular box).

**Figure 9:**
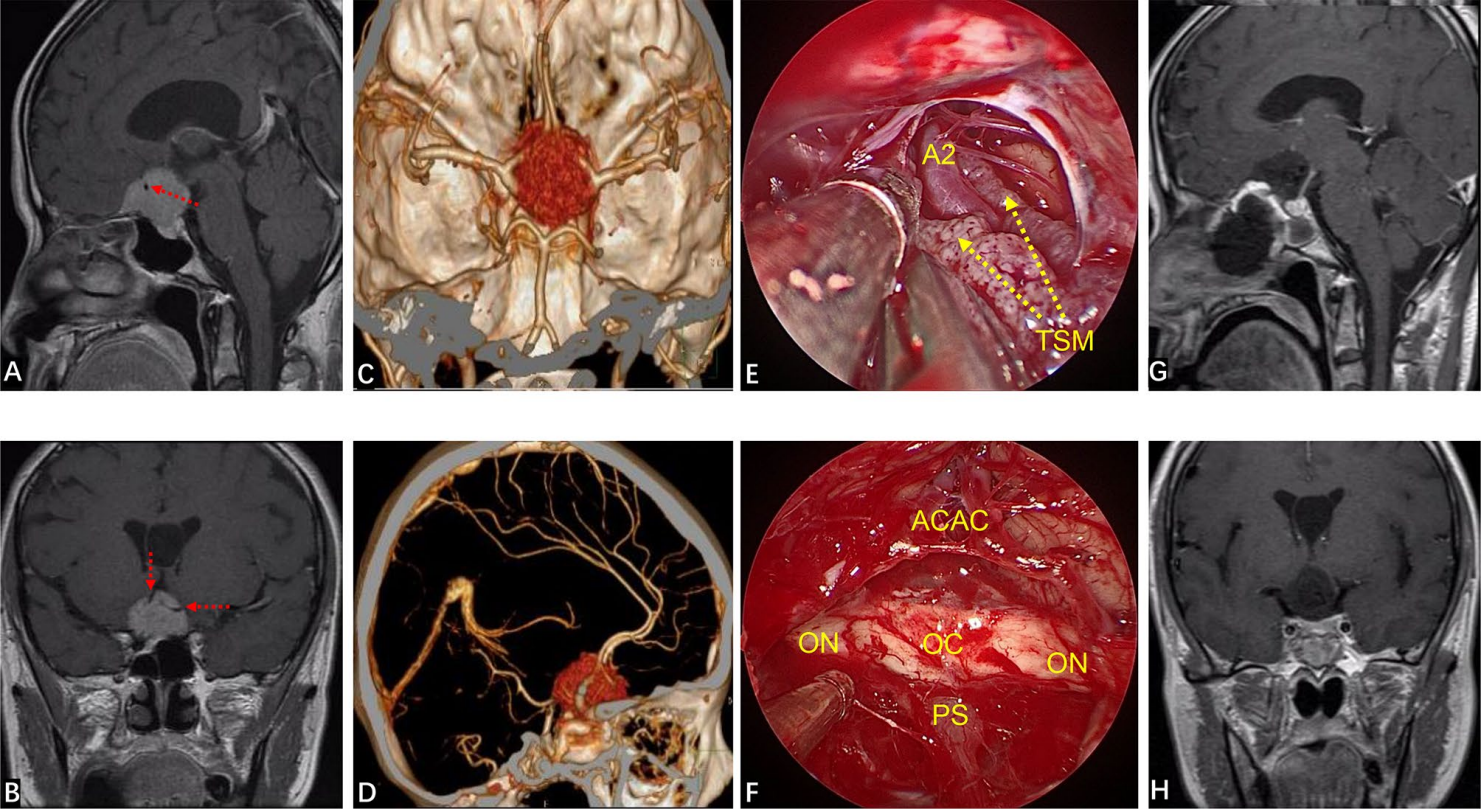
(**A**,**B**) Preoperative contrast-enhanced MRI of the sellar region showed the ACA (red dotted arrow) was wrapped by the tumor at 360°. (**C**,**D**) Preoperative computed tomography angiography (CTA) examination indicated that the tumor to be completely wrapped around the ACAC and was closely related to the ICA. (**E**) This intraoperative image shows the tumor to be completely wrapped around the A2 branch of the ACA. (**F**) After the tumor had been removed completely, the optic nerve, pituitary stalk and ACAC was well protected. (**G**,**H**) At POD3, contrast-enhanced MRI of the sellar region showed complete resection of the tumor and that the optic nerve, pituitary stalk and pituitary gland were intact.

**Figure 10:**
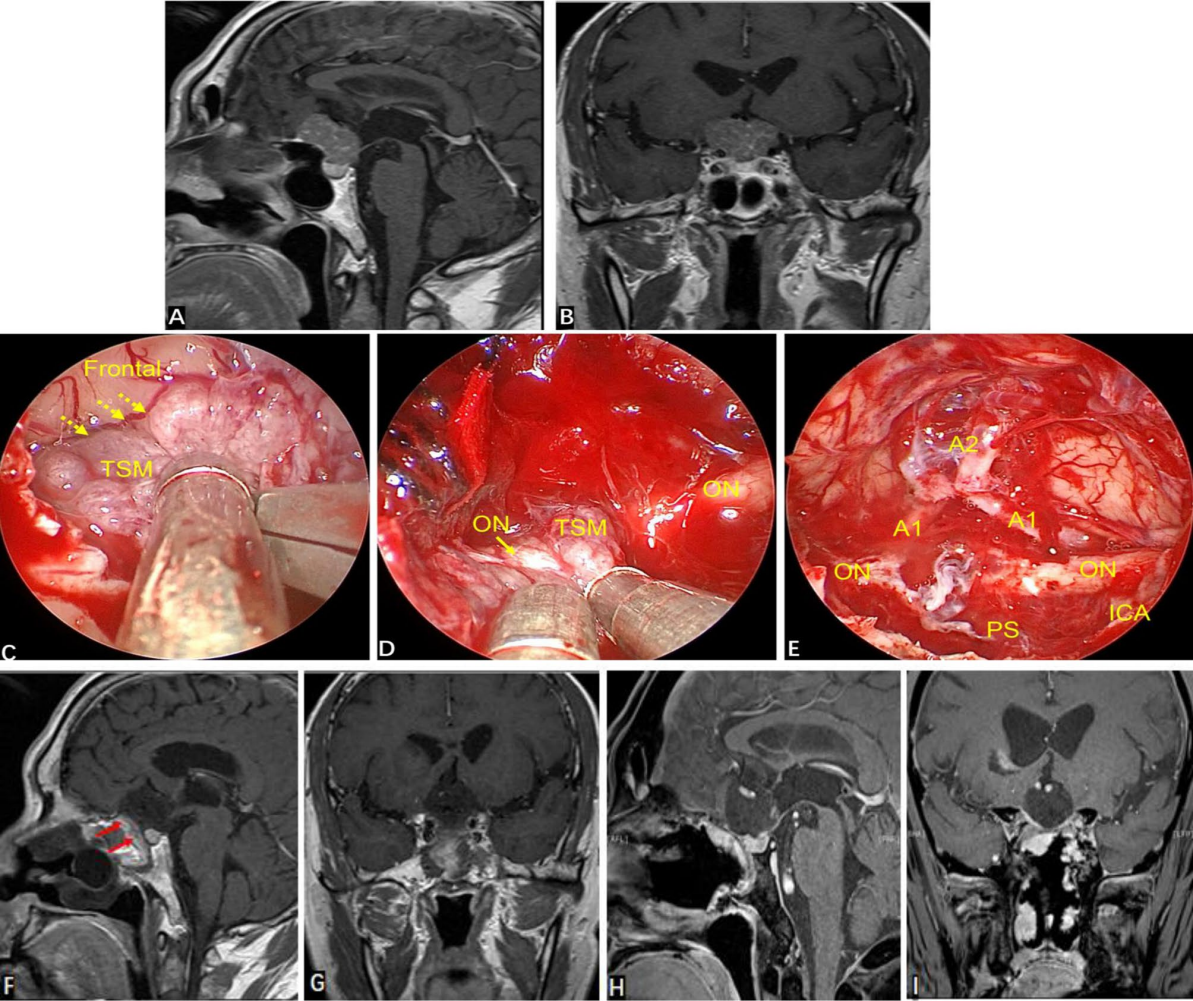
(**A**,**B**) Preoperative contrast-enhanced MRI of the sellar region indicated that the tumor was closely related to the ACA and ICA. (**C**) The tumor had no arachnoid plane, and adhered widely to the frontal lobe (yellow dotted-line arrow). (**D**) The right optic nerve had been invaded, and adhesion with the tumor was very severe. (**E**) After total resection of the tumor, invasion of the right optic nerve was revealed. (**F**,**G**) Contrast-enhanced MRI of the sellar region at POD3 suggested total resection of the tumor, and the VNF healed well (red solid-line arrow). (**H**,**I**) Six months after surgery, contrast-enhanced MRI of the sellar region showed no sign of residual tumor or recurrence.

Among our patients, 38 (95.0%) achieved GTR (Simpson grade I), and 2 (5.0%) achieved NTR. The tumor of one patient was linked inextricably to the A2 branch of the ACA, and resulted in a small residual tumor (Fig. 8). In another patient, the tumor adhered closely to the left optic nerve and ICA, resulting in some tumor mass remaining at the left ICA. Although there was no obvious tumor remaining according to postoperative MRI, it could not be termed GTR (Fig. 5). GTR was achieved in all other patients. In our series, tumor diameter, arachnoid plane and optic-canal invasion were not significant parameters for GRT (Table 3). However, adhesion between the tumor and blood vessels (pay attention to adhesion rather than wrapping) is an important factor affecting GTR. In some tumors, even though some large blood vessels are surrounded by the tumor at 360°, there is no adhesion between the tumor and blood vessels. Hence, GTR can be achieved as long as the tumor is removed piece-by-piece. The operative field of view is clear under endoscopy, local structures are enlarged, and multi-angle observation can protect these blood vessels (Fig. 9). Thirty-four patients (85.0%) had a complete arachnoid plane, and six patients (15.0%) lacked an arachnoid plane, where the tumor adheres to nerves and the frontal lobe hampered separation, posing a huge challenge, but for a team with rich experience in the EEEA, it did not affect GTR of the tumor (Figs. 3B–D, 10).

**Table 3:** Characteristics related to GTR.

Characteristic	Number of patients	Number of GTR achieved (%)	P
**Maximum diameter (mm)**			1.0
≤ 24.1	25	24 (96.0%)	
> 23	15	14 (93.3%)	
**Invasion of optic canal**			1.0
Yes	6	6 (100%)	
No	34	32 (94.1%)	
**Arachnoid plane**			0.281
Present	34	33 (97.1%)	
Absent	6	5 (83.3%)	
**Adhesion with important vessels**			0.001
Present	2	0 (0%)	
Absent	38	38 (100%)

### Surgical complications

In recent years, with improvements in the technology of skull-base reconstruction (especially application of VNF), the prevalence of CSF leakage after endoscopic transnasal surgery has been reduced considerably. Researchers have reported that the prevalence of CSF leakage after application of a VNF is < 5%^33,34^. With the accumulation of experience, our research-team’s concept of skull-base reconstruction is improving and updating constantly. In our early surgical procedures, reconstruction of the skull base was undertaken with only the VNF. In 2017, we began to use the in situ bone flap from the sellar region to suprasellar region for restoration (Fig. 2). The difference compared with using only the VNF was significant (p < 0.05) (Table 4). However, the skull-base bone itself is relatively thin, and forming the bone window in situ at one time is difficult, and bone fracture and non-formation of bone often occur. For such patients, we use autogenous thigh fat and fascia lata to reconstruct the skull base. Application of an autogenous in situ bone flap can not only restore the original anatomic structure of the skull base, but also quickly change the high flow CSF leakage into low flow exudate, which further reduce the incidence of CSF leakage. In our study, drainage of lumbar cisterns was not undertaken routinely postoperatively. Only three patients (7.5%) had CSF leakage postoperatively, and we used only the VNF to reconstruct the skull base in them.

**Table 4:** Prevalence of CSF leakage after different types of skull-base reconstruction.

Method	No CSF leakage	CSF leakage	Total	CSF leakage (%)
Vascularized nasoseptal flap	10	3	13	23.1
Vascularized nasoseptal flap and in situ bone flap	23	0	23	0
Total	33	3	36^a^	8.3
Statistical analysis		P = 0.04		

^a^Autogenous thigh fat and fascia lata were used to reconstruct the skull base in four patients.

Nasal complications after endoscopic transnasal surgery occur, but few scholars have paid attention to them (especially olfactory disorders). Different results have been garnered on the prevalence of olfactory disorders after endoscopic surgery. Charalampaki et al. analyzed the complications of 150 cases of endoscopic resection of pituitary adenomas. They found that the prevalence of hyposmia was 10%, and the prevalence of anosmia was 2%^35^. David et al. undertook a prospective study on the olfactory effects of endoscopic transnasal surgery for pituitary tumors. For patients with normal olfaction before surgery, 1.5% of patients developed hyposmia after surgery, and 1.5% of patients developed anosmia^36^. In our study, eight patients (20.0%) developed hyposmia postoperatively. Of these eight cases, five patients had nasal irrigation in the early postoperative period, four patients had olfaction return to normal, and one patient had a significant improvement in olfaction. Three patients (7.5%) developed long-term hyposmia, and two of them had CSF leakage and infection after surgery. The study of olfactory dysfunction after endoscopic transnasal surgery has been reported, but the pathogenesis is incompletely understood. It may be related to: VNF preparation; injury to the nasal epithelium, olfactory conduction pathway and olfactory center by surgery; inflammation in the nasal cavity after surgery; obstruction of sinus drainage caused by adhesion of the nasal cavity^35,37,38^. Brian et al. hypothesized that attention should be paid to olfaction and mucosal protection through careful surgical procedures but that, ultimately, these measures do not reduce the prevalence of olfactory disorders^39^. In our study, two patients had CSF leakage and infection after surgery that was associated with long-term hyposmia. The olfactory function of five patients with hyposmia was improved to different degrees after nasal irrigation. Therefore, considering the inflammatory reaction in the operative area of the nasal cavity, adhesion and scar formation in the nasal cavity are the main causes of olfactory dysfunction. Therefore, for patients with postoperative hyposmia without CSF leakage, we recommend early nasal irrigation.

Other complications included bleeding of the A2 branch of the ACA in one patient during surgery due to improper blunt traction. This led to CNH infarction on POD5 (Fig. 4). For the transnasal approach of TSM, the safe surgical strategy is to be able to achieve sharp separation of tumor and vascular adhesion under direct vision. For large tumors, the basis of sharp separation under direct vision is to fully resect the tumor piece-by-piece and avoid blind tugging in the subdural stage.

### Limitations

This study has some limitations since it was designed as a nonrandomized retrospective study and the incomplete exclusion of potential selection biases.

## Conclusions

The EEEA is a safe, reliable minimally invasive surgical method for TSM removal. It can transform a deep skull-base tumor into a superficial convex tumor, and the invaded bone and dura mater can be resected simultaneously. It can deal with the blood supply of the tumor at an early stage without retraction of the optic nerve during surgery, and has obvious advantages in the protection of perforating vessels, which can promote GTR. Compared with traditional craniotomy, the EEEA may have better visual outcome, but carries the risk of CSF leakage. The EEEA has general limitations based on tumor diameter and whether the tumor encases a large blood vessel. Hence, skull-base endoscopy has great potential for application.
